# Genome Sequences of Alatato and Audell, two Phages that infect *Arthrobacter globiformis* and have Different Life Cycles

**DOI:** 10.17912/micropub.biology.001786

**Published:** 2025-12-03

**Authors:** Sarah E Robinson, Erin Stanford, Shilen Abraham, Rebecca Aguilera, Nihal S Anantoji, Marco Barillas, Ryan Barstys, Emily Benson, Kayce R Boucher, Adrianna M Chacko, Niveditha Chandrakanth, Angel Chau, Angela Chen, John Coleman, Luong Tri Duc, Olivia Godfrey, Sasha N Hoang, Medhana Kethamreddy, Nisarg Kumar, Bao Ngan Le, Gregory T Lin, Kim B Nguyen, Madhura Pandit, Kelie D Shah, Mohini Sharma, Sophia Singh, Josh Smith, Lucy V Thomas, Alvin Varghese, Ian Wells, Richard S Pollenz

**Affiliations:** 1 Molecular Biosciences , University of South Florida, Tampa, Florida, United States

## Abstract

Based on gene content similarity, Alatato and Audell are grouped to phage clusters FB and FR, respectively. Alatato has a 39,780bp genome and 71 genes of which 34 have a predicted function. Alatato is predicted to be temperate based on the identification of an immunity cassette with genes that encode a tyrosine integrase, helix-turn-helix DNA binding proteins and an excise. Audell has a 42,187bp genome and 67 genes of which 32 have a predicted function with five enzymes involved in thymine synthesis. Putative lysis cassettes with genes encoding endolysins and transmembrane proteins were identifiable in both phages.

**Figure 1. Plaques and particles of bacteriophages Alatato and Audell:**
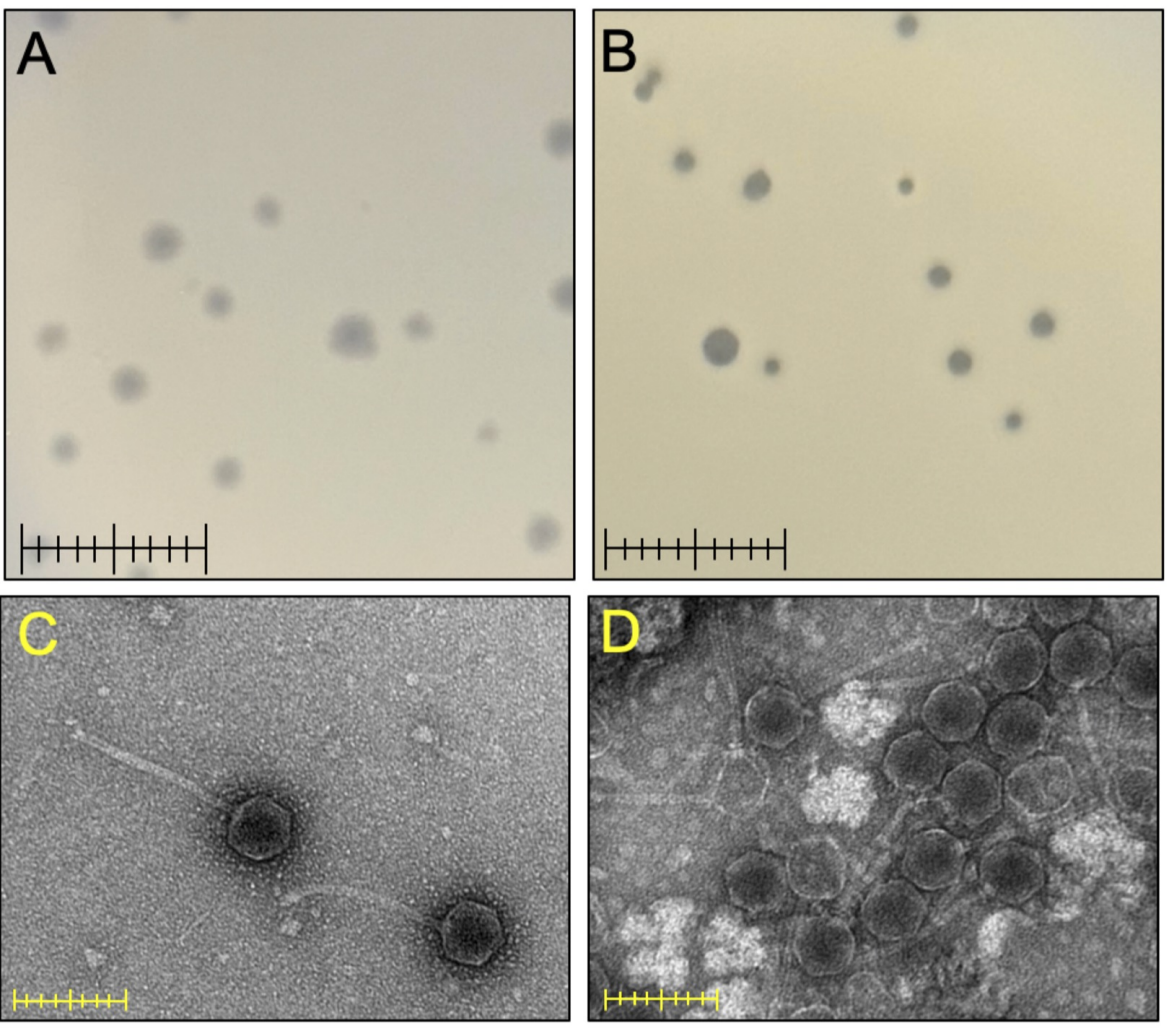
Plaque sizes of Alatato (A) and Audell (B). Ruler = 1cm. Transmission electron micrographs of Alatato (C) and Audell (D) stained using UryanLess (Electron Microscopy Sciences). Ruler = 200nm.

## Description

There are an estimated 10 ^31^ phage particles in the biosphere (Rohwer et al., 2014) and to date the SEA- PHAGES program (Jordan et al., 2014) has isolated and annotated over 5,500 novel actinobacteriophages that are archived in the Actinobacteriophage Database at PhagesDB.org (Russell and Hatfull, 2017). Phages offer a vast repository of uncharacterized genes and the isolation and annotation of evolutionarily diverse phages can help advance the field of phage genomics and the application of phages to the fields of agriculture, food safety, and medicine (Shield et al., 2021; Wang and Zhao, 2022).

Alatato and Audell were isolated from ~15g moist soil samples taken from different areas of Tampa, FL. GPS coordinates are shown in Table 1. Soil samples were mixed with equal parts peptone-yeast calcium media (PYCa), shaken at 250rpm for 2 hr. at 25 ^o^ C and filter sterilized (0.2µm PES). 500ul of the sterile soil filtrates was mixed with 250ul of saturated *Arthrobacter globiformis* NRRL B-2979 and plaque assays performed by mixing the sample with 5ml of PYCa Top Agar and plating on PYCa agar plates. Plates were incubated at 30 ^o^ C for 18-24 hrs. A single plaque that was separated a minimum of 2cm from others was picked with a sterile 1000ul pipette tip, resuspended in 100ul of Phage Buffer (PB; 10mM Tris pH 7.5; 1mM CaCl2; 10mM MgSO4; 68mM NaCl), vortexed and then serial diluted in PB. After a 10-minute incubation at room temperature, 10ul aliquots were used from each serial dilution to complete plaque assays as described above. A plaque was picked from the new set of plates, serial diluted and plated as above to assure a clonal population with uniform plaque morphology. High titer lysates (> 5 x 10 ^9^ PFU/ml) were prepared from these plates and genomic DNA was isolated using the Wizard DNA clean-up kit (A7280; Promega). Genomic DNA was used to create sequencing libraries with the NEB XLEP-P1 kit. Sequencing was performed by the Pittsburgh Bacteriophage Institute and the library run on an Illumina NextSeq 1000 instrument. Raw reads were trimmed with cutadapt 4.7 (using the option: –nextseq-trim 30) and filtered with skewer 0.2.2 (using the options: -q 20 -Q 30 -n -l 50) prior to assembly. Sequencing data for each phage is presented in Table 1. The resulting sequences were assembled using Consed (v29.0) with Unicycler (v5.0) and contigs checked for completeness, accuracy, and genome termini (Gordon et al., 1998; Russell, 2018). Default parameters were used for all software. The genomes of both Alatato (‘5-TGCCGCCCGGTA) and Audell (5'-TTTACGGCAT) have 3' sticky overhangs and were bioinformatically linearized such that base 1 is assigned in accord with other *Arthrobacter* phages (Russell, 2018). Both genomes were auto-annotated using DNA master (v5.23.6) (Pope and Jacobs-Sera, 2018) and the genes then manually validated for starts and functional calls. GeneMark (v2.5) (Besemer and Borodovsky, 2005) and Glimmer (v3.02) (Delcher et al., 2007) were utilized to assess start sites and coding potential. Starterator (v601; github.com/SEA-PHAGES/starterator) to summarize the starts across each family of phage genes. Evidence to support a gene product function was collected using HHpred (databases: PDB_mmCIF70_30_Mar; Pfam-A_v37; NCBI_Conserved_Domains(CD)_v3.19) (Söding, 2005; Marchler-Bauer, 2015), and NCBI BLAST (BLAST+2.13) (Altschul et al., 1990). Putative transmembrane domains (TMD) were identified using Deep TMHMM (v1.0.24) (Hallgren et al., m2022) and TOPCONS (v2.0) (Tsirigos et al, 2015). Information on each phage regarding isolation, characterization, and gene content is archived in Phamerator (Actino_draft database v578; Cresawn et al., 2011), and the Actinobacteriophage Database at PhagesDB.org ( https://phagesdb.org/phages/Alatato/ and https://phagesdb.org/phages/Audell/ ).

Alatato and Audell were assigned to clusters FB and FR, respectively based on gene content similarity of at least 35% to phages in the Actinobacteriophage database, PhagesDB ( https://phagesdb.org ) (Pope et al., 2017; Russell and Hatfull, 2017). Both phages are siphoviruses as determined by negative stain transmission electron microscopy and have icosahedral capsids and flexible tails (measurements presented in Figure 1). Alatato produces cloudy plaques ranging from 1 mm to 2.5 mm (Figure 1). The establishment of lysogeny by phage Alatato is predicted based on the identification of a putative immunity cassette inclusive of and flanked by genes encoding a tyrosine integrase (reverse gene *29* ) and an excise (forward gene *35* ). Within this flanked region, Alatato reverse gene *33* and forward genes *32* and *34* all encode helix-turn-helix (HTH) DNA binding domain proteins that likely function as repressors based on high probability HHpred hits to the lambda CI repressor PDB 1LMB_3 (Beamer and Pabo, 1990). All of the other FB phages appear to have an immunity cassette in a similar region of the genome but there is some diversity in gene content and organization. While all contain an integrase grouped to the same pham as Alatato, this gene is found in the forward direction in phages Kumotta and MargaretKali. All FB phages encode a protein grouped in the same pham as Alatato HTH proteins gp31 and gp33, but only phage Shoya contains a forward gene encoding a protein orthrolog to Alatato gp32. Sarge and Shoya encode a protein grouped in the same pham as Alatato gp34, while the other FB phages have a different gene in this region that also encodes an HTH protein but is grouped to a different pham based having <34% identity Alatato gp34. Thus, all FB phages have genes that encode at least three distinct HTH proteins and since all of the genes are predicted to encode repressors, it is not possible to identify which might serve as the immunity repressor or cro protein without additional wet lab experiments. Alatato also contains a putative lysis cassette with gene *21* encoding an endolysin flanked upstream by gene *19* encoding a protein with a single transmembrane domain (TMD) and downstream by gene *22* encoding a putative 2TMD holin.

Phage Audell shares 67%, 76% and 72% gene content to FR phages Neuvillette (draft), TMaxx (draft) and AnnabelLee, respectively. Audell produces clear plaques ranging from 1 mm to 2.5 mm (Figure 1) and is predicted to be lytic based on the absence of genes associated with lysogeny. Audell has a lysis cassette organization similar to Alatato with gene *24* encoding an endolysin flanked upstream by gene *23* encoding a 1TMD protein and downstream by gene *25* encoding a 2TMD holin protein similar to Alatato. Although the function of these proteins in lysis has not been evaluated in Arthrobacter phages, the lysis cassette organization is different from phages that infect the Gram positive *Gordonia rubripertincta* NRRL B-16540 strain, where the TMD encoded proteins are found downstream of the endolysin (Pollenz et al., 2022; McGarrah et al., 2023). Audell contains several genes that encode enzymes predicted to be involved in thymine synthesis including: dUTPase (gene *33* ), dihydrofolate reductase (gene *41* ), deoxycytidylate deaminase (gene *43* ), thymidylate kinase (gene *50* ) and ribonucleotide reductase (gene *57* ). These enzymes may be involved in thymine hypermodification that protect the phage genome from host defense systems (Lee et al., 2021).

**Data availability**

Alatato whole Genome Shotgun project has been deposited in DDB/ENA/GenBank under the accession no. PV915846 and SRX28150552 . Audell whole Genome Shotgun project has been deposited in DDB/ENA/GenBank under the accession no. PV915817 and SRX28150564 . The versions described in this paper are the first version.

**Table 1.** **Summary of relevant sequencing information, isolation parameters, genome characteristics and morphology measurements of bacteriophages Alatato and Audell**

**Table:** 

Parameter	Bacteriophage	
phagesDB link	Alatato	Audell
Location of sample collection, GPS Coordinates	Tampa, FL. 28.06 N, 82.4180 W	Tampa, FL. 27.9275 N, 82.1655 W
Single-end Read Size	100bp	100bp
Single-end Reads, #	2.6 x 10 ^6^	2.9 x 10 ^6^
Shotgun Coverage	6,536	6,874
Genome Size (bp)	39,780	42,187
G/C content (%)	63.8	58.6
Total Protein Encoding Genes	71	67
Genes with Functional Annotation	34	32
tRNA Genes	0	0
Cluster	FB	FR
Capsid Diameter	49.7 nm +/- 3.4 nm (n = 10)	50.5 nm +/- 2.8 nm (n = 12)
Tail Length	255 nm +/- 5.5 nm (n = 10)	230 nm +/- 4.3 nm (n = 12)
